# Study on the adsorption kinetics of natural dye by colored paper fiber raw material

**DOI:** 10.3389/fchem.2026.1868454

**Published:** 2026-09-09

**Authors:** Tinghui Zhang, Zhenhua Yu, Shuwei Guo, Chunhua Yang, Baoshan Yue, Jiao Xie, Zhihua Liu, Hao Wang, Han Zheng, Ying Zhang, Tingting Yu, Yadian Xie, Yi Dai

**Affiliations:** 1 School of Chemical Engineering, Guizhou Minzu University, Guiyang, China; 2 Technology Center, China Tobacco Yunnan Industrial Co., Ltd., Kunming, China; 3 Kunming Cigarette Factory, Hongyunhonghe Tobacco (Group) Co. Ltd., Kunming, China; 4 School of Fine Arts, Guizhou Minzu University, Guiyang, China

**Keywords:** adsorption kinetics, colored cigarette paper, fiber raw materials, natural dyes, online UV-Vis spectroscopy

## Abstract

To ensure stable quality of colored cigarette paper dyed with natural dyes, real-time kinetic data of dye adsorption onto cellulosic fibers remains insufficient due to limitations of traditional offline sampling methods. In this work, brown natural dye was used as the adsorbate, and an online UV-Vis spectral monitoring system was developed to achieve continuous, non-destructive detection of the full adsorption process without intermittent sampling. The effects of initial dye concentration and fiber type on the adsorption behavior and mechanism were systematically investigated. All experiments were conducted at 20 °C with a pulp consistency of 0.2% (w/v), and the initial dye concentration ranged from 10 to 80 mg/L. Pseudo-first-order (PFO) was applied for data fitting. The adsorption process exhibits two successive stages: a water absorption-swelling induction period and a dye adsorption stage. A competitive adsorption mechanism between water molecules and dye macromolecules on fiber surfaces is proposed to explain the induction period. The induction time decreases exponentially with increasing initial dye concentration, following (y = 6,998*e^−0^·^047^ˣ) with (R^2^ = 0.987). Within 30–80 mg/L, the equilibrium adsorption capacity (q_e_) increases linearly with dye concentration. For hemp pulp fiber at 80 mg/L, (q_e_) reaches 1.1184 mg/g, the PFO rate constant (k_1_) is 0.0012 s^-1^ with (R^2^ = 0.9980). The PFO model shows better fitting performance for all systems, indicating a diffusion-controlled physisorption process. Hardwood pulp reaches equilibrium at ~1,500 s with the fastest initial rate, while softwood and hemp pulp require over 10000 s. This work provides fundamental kinetic data and theoretical support for formula design and process optimization of online dyeing for colored cigarette paper.

## 1 Introduction

Cigarette paper is an important component of the “three papers and one rod” used as auxiliary cigarette materials (Peng et al., 2014; Zhang et al., 2024; Zheng et al., 2021). It is mainly composed of fibers, fillers, and functional additives (Luo et al., 2015; Shen et al., 2014; Zhou et al., 2011). It is generally white in color (Zhang et al., 2021). In recent years, colored cigarette paper, with its distinctive and individualized characteristics, has endowed cigarettes with unique features and visual appeal (Xiang, 2009). To ensure the harmony between the aroma and taste of additives in colored cigarette paper and the sensory characteristics of cigarettes, as well as the safety of the additives, tobacco extracts are widely used as colorants in colored cigarette paper in China (Chen et al., 2017; Yang et al., 2009). In actual production, according to the properties of the colorants and the design requirements of colored cigarette paper, two coloring methods are generally adopted (Chen et al., 2017; Gu et al., 2018; Peng et al., 2014): One method is to produce white cigarette paper (base paper) first, followed by offline coating with tobacco extract, drying, and rewinding. The other is to directly add tobacco extract for coloration during the sizing stage in the latter half of cigarette paper production, followed by drying and winding.

In the online coating process for colored cigarette paper, paper color is affected by formulation components such as tobacco extract dyes at different proportions or from different raw material batches, as well as fibrous raw materials (Chen et al., 2017; Gu et al., 2018; Wang et al., 2015; Yang et al., 2009). As a result, the hue uniformity and stability of the produced colored cigarette paper, both within and between batches, still need improvement. At the same time, the residence time of white cigarette paper (base paper) in the dye solution is short. In addition, the adsorption behavior of the selected colorants on fibrous raw materials and other paper components remains unclear. This makes it difficult for production personnel to monitor and control colorant concentration during manufacturing, further increasing intra-batch non-uniformity and inter-batch color differences in colored cigarette paper. Regarding the fiber formulation of colored cigarette paper, each type of colored paper is typically prepared from a blend of at least two kinds of fibrous raw materials (Huang, 2005; Ma and Cao, 2015). Because fiber type and chemical composition differ, their dye adsorption capacities must also vary (Lin et al., 2024; Maaß et al., 2024; Parameswaranpillai et al., 2023; Pulkkinen et al., 2009; Vibert et al., 2024). Fiber structure determines its main properties. During pulp refining, fibrillation changes the surface and interfacial structure of fibers, exposing a large number of hydroxyl groups and enhancing the adsorption of aqueous dye particles by the fibers (Wei, 2022). In terms of physical properties, fiber specific surface area, pore structure, and surface charge are also important parameters affecting the adsorption performance of pulp fibers in aqueous solution (Jiang et al., 2022; Kuang et al., 2014). Different types of pulp fibers vary greatly in specific surface area, pore characteristics, and charge properties, and the proportions of their chemical components, including cellulose, hemicellulose, and lignin, also differ (Ma and Cao, 2015). Most existing studies focus on the adsorption of synthetic dyes on pulp fibers. Nevertheless, synthetic dyes are banned for colored cigarette paper due to strict food-grade safety requirements. Consequently, systematic research on the adsorption kinetics and thermodynamics of natural dye remains insufficient. Featuring excellent biocompatibility and sensory performance, natural dyes have great application potential for colored cigarette paper. In terms of detection methods, most conventional studies adopt offline batch sampling. This approach is unable to capture the real-time dynamic changes during the whole adsorption process. More importantly, natural dyes present relatively low dye uptake on fibers, and the inherent errors of offline detection will be further amplified, leading to inaccurate and unreliable experimental data.

To address the lack of real-time kinetic data and unclear adsorption mechanism of natural dyes on pulp fibers, an online monitoring platform for the whole adsorption process is constructed in this work based on dynamic spectral detection technology, realizing uninterrupted real-time tracking of dye concentration changes. Using brown natural dye as the adsorbate, the effects of initial dye concentration and fiber type on the adsorption behavior are systematically investigated. Combined with pseudo-first-order kinetic model fitting, the adsorption kinetic characteristics and underlying mechanism of different pulp fibers are revealed. Meanwhile, the influence trends of beating degree and fiber blending ratio on the equilibrium dye adsorption capacity are preliminarily explored as supplementary results. This study aims to provide basic kinetic data and theoretical reference for fiber formula optimization and precise process regulation of online dyeing for colored cigarette paper.

## 2 Materials and methods

### 2.1 Chemicals and samples

The fibrous raw materials, including long-fiber pulp (Canada), short-fiber pulp (Brazil), and hemp pulp (France), were all obtained from Yunnan Hongta LanYing Paper Co., Ltd. The brown dye was also provided by Yunnan Hongta LanYing Paper Co., Ltd.

Conventional analytical reagents were purchased from Aladdin Reagent Co., Ltd. Ultrapure water was prepared in the laboratory.

### 2.2 Apparatus and operations

The equipment used in this study included a PFI mill (IMT-MJ01, Dongguan Intenson Precision Instrument Co., Ltd., China), a high-speed disperser (SDF, Laizhou Gerui Machinery Co., Ltd., China), a magnetic stirrer (SP18425, Xiamen Xingruida Automation Equipment Co., Ltd., China), a UV–Vis spectrophotometer (Agilent 8453, Agilent Technologies, United States), a pulp disintegrator (97015, L&W, Sweden), a beating degree tester (PN-SDJ100, Hangzhou Pinxiang Technology, China), a peristaltic pump (Lead Fluid WT600F, China), peristaltic tubing (fluid silicone tubing, inner diameter 2 mm, wall thickness 1 mm), and a flow cuvette (optical path lengths of 10, 1, and 0.1 mm, Agilent Technologies, United States).

### 2.3 Experimental methods

#### 2.3.1 Raw material treatment and online monitoring of dye concentration

1. Treatment of pulp fibers

Different types of pulp board were first subjected to preliminary water absorption and swelling. They were then refined using a PFI mill to prepare pulp fibers with different beating degree gradients. The refined pulp fibers were dewatered, sealed for equilibration, and stored for later use.

2. Online monitoring of dye particle adsorption by pulp fibers

The online UV-Vis spectroscopy was applied to monitor the dye adsorption process. Distilled water was used as blank for background correction. The initial absorbance (A_0_) and time-dependent absorbance (A_t_) of dye solution were measured sequentially, and the dye adsorption capacity was calculated from absorbance variation. The detection wavelength was set at 475 nm, the total detection time ranged from 1,800 to 3,600 s, and the sampling interval was 10–30 s. In all tests, the solution volume was 100 mL, the system temperature was kept at 20 °C combined with the actual industrial production conditions, and the natural solution pH was 6.5. The oven-dried pulp fiber consistency was 0.2%, and the peristaltic pump worked at 3 mL/min. Data were recorded every 30 s, and the first reading was taken as (A_0_). All quantitative adsorption equilibrium experiments (e.g., equilibrium adsorption capacity) were performed in triplicate, and the corresponding results are expressed as mean values with standard deviations. For real-time online adsorption kinetic tests, continuous time-resolved dynamic monitoring was conducted, and representative kinetic curves are presented in the figures.

#### 2.3.2 Calculation of dye concentration in solution

A series of standard dye solutions was prepared, with concentrations of 20, 30, 40, 50, 60, 80, and 100 mg/L, respectively. Using the detection device equipped with a flow cuvette, the absorbance of these solutions was measured at 475 nm. The mathematical relationship between absorbance and concentration was then obtained as follows:

$$A=0.0040\left(\pm 0.0039\right)+0.0029\left(\pm 0.0002\right)C\;\left(n=7,R^{2}=0.999\right)$$ (1)

where C is the concentration of the dye solution, in mg/L.

Therefore, according to Equation 1, the dye content in the solution can be determined from the relationship between absorbance and concentration.

## 3 Results and discussion

### 3.1 Establishment of an online spectroscopic monitoring platform for dye adsorption by pulp fibers

**FIGURE 1:**
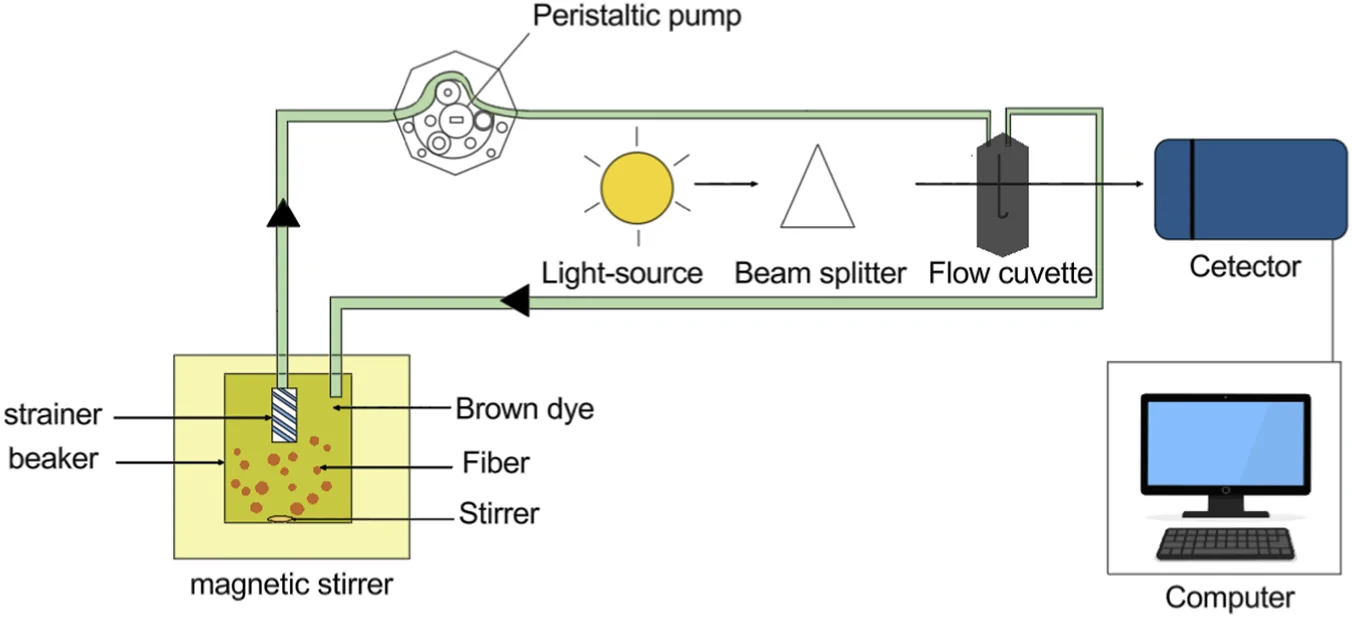
Schematic diagram of an online monitoring platform for pulp fiber adsorption of dyes.

**FIGURE 2:**
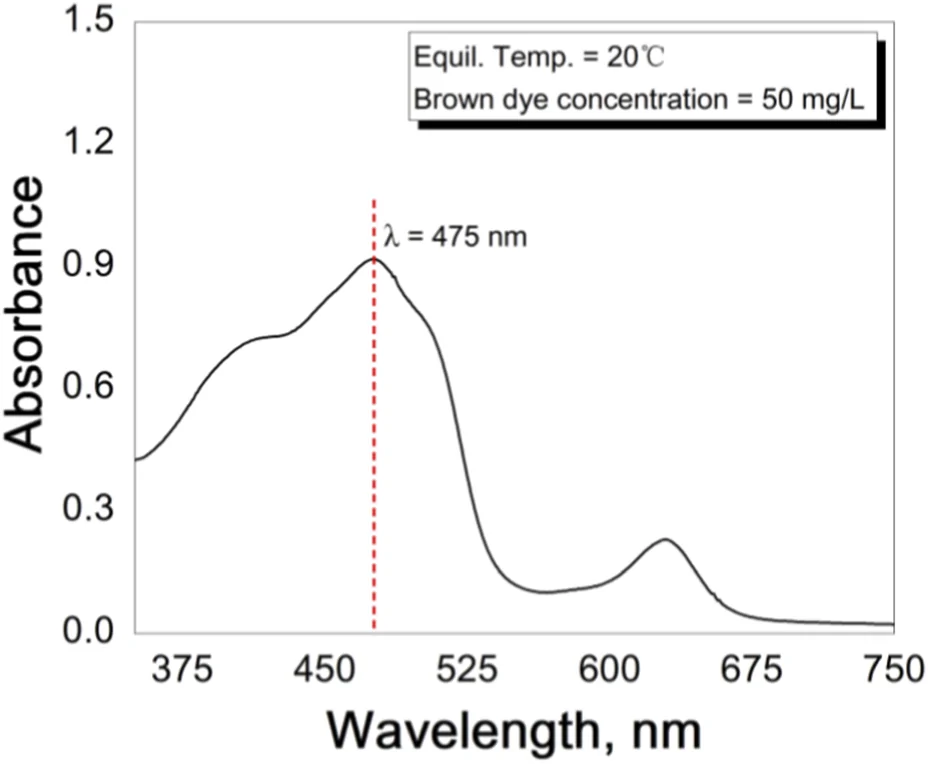
Ultraviolet-visible spectrum of brown dye adsorbed by pulp fibers.

Figure 1 shows the online spectroscopic monitoring platform used to investigate the dye adsorption process of pulp fibers. In this device, the dye solution in the reaction vessel was circulated by a peristaltic pump through a flow cuvette in a UV-Vis spectrophotometer for continuous detection. A 400-mesh filter wrapped around the inlet of the tubing was used to remove fine fibers and impurities from the solution, thereby preventing blockage of the pipeline and minimizing spectral baseline drift. Figure 2 shows the UV-Vis absorption spectrum of brown dye adsorbed by pulp fibers. As shown in the figure, the brown dye exhibited absorption in the visible region (400–760 nm), with a maximum absorbance at 475 nm. A small amount of soluble lignin from some pulp fiber samples produced a background signal at 285 nm, but this did not interfere with the spectral measurement. Therefore, in the subsequent experiments, the adsorption behavior and mechanism of brown dye on pulp fibers, fillers, or cigarette base paper were investigated by monitoring the change in absorbance at 475 nm.

### 3.2 Validation and optimization of online UV-Vis monitoring system

All pre-experiments in this section were conducted to verify the stability of the self-developed online UV-Vis monitoring system and determine the optimal operating parameters, so as to ensure the accuracy and reliability of subsequent adsorption detection data.

#### 3.2.1 Optimization of peristaltic pump and pipeline

The peristaltic pump has two main functions in the online detection platform. It drives circulating flow of aqueous solution between the reaction cell and pipelines to maintain a constant concentration of pulp fibers. Meanwhile, it delivers stable dye solution to the flow-through cuvette for real-time UV-Vis spectral measurement. The fluid delivery of a peristaltic pump is realized by periodic compression and release of elastic tubes.

**FIGURE 3:**
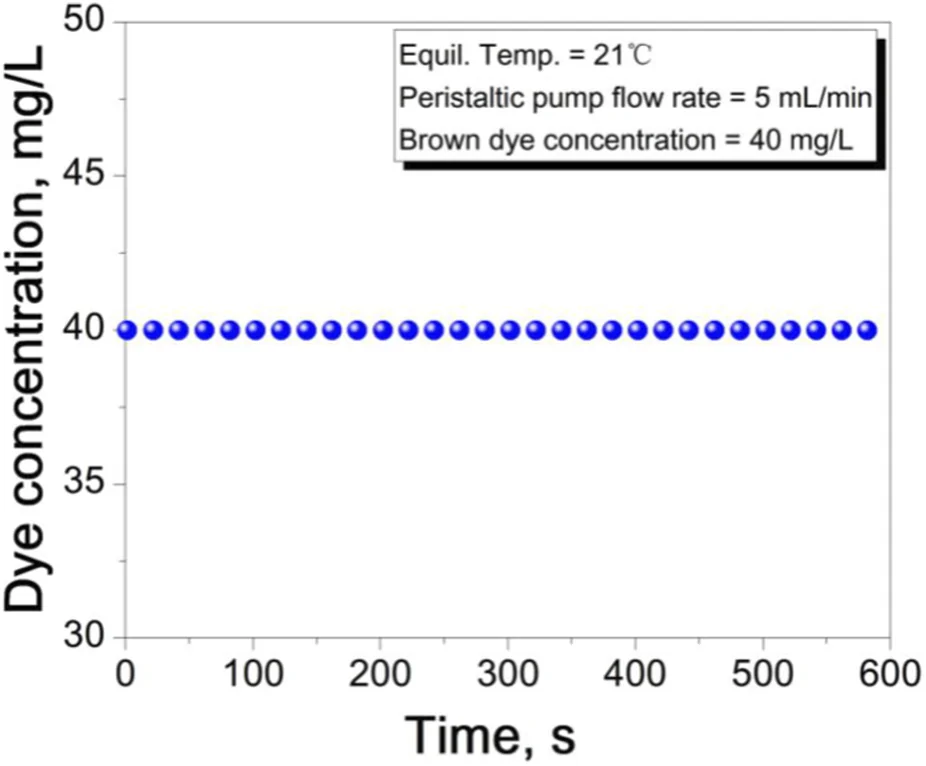
Stability verification of liquid delivery by peristaltic pump.

Four types of commercial peristaltic pumps, namely Pure Fluid, Baoding Qili, Gilson and Leifu WT600F, were tested comparatively. The results indicated that most pumps produced obvious flow pulsation, which caused drastic fluctuation of absorbance values. This defect is unfavorable for high-frequency kinetic detection. By contrast, the Lead Fluid WT600F presented minor flow pulsation and stable absorbance readings within 600 s (Figure 3). Thus, this model was selected as the fluid delivery device for the whole system.

The circulating tubes connecting reaction and detection units were also optimized. Hard tubes cannot achieve precise flow control, while tubes with too small inner diameter tend to adsorb dye on the inner wall, leading to detection errors and difficulty in post-experiment cleaning. After multiple tests, a highly flexible silicone tube with an inner diameter of 2 mm and a wall thickness of 1 mm was confirmed to have the best performance. In addition, small-size filters were installed at the pipeline inlet to mitigate fiber hanging. This treatment guarantees full dispersion of pulp fibers and sufficient contact between fibers and dye molecules.

#### 3.2.2 Effect of pulp fiber concentration on detection stability

**FIGURE 4:**
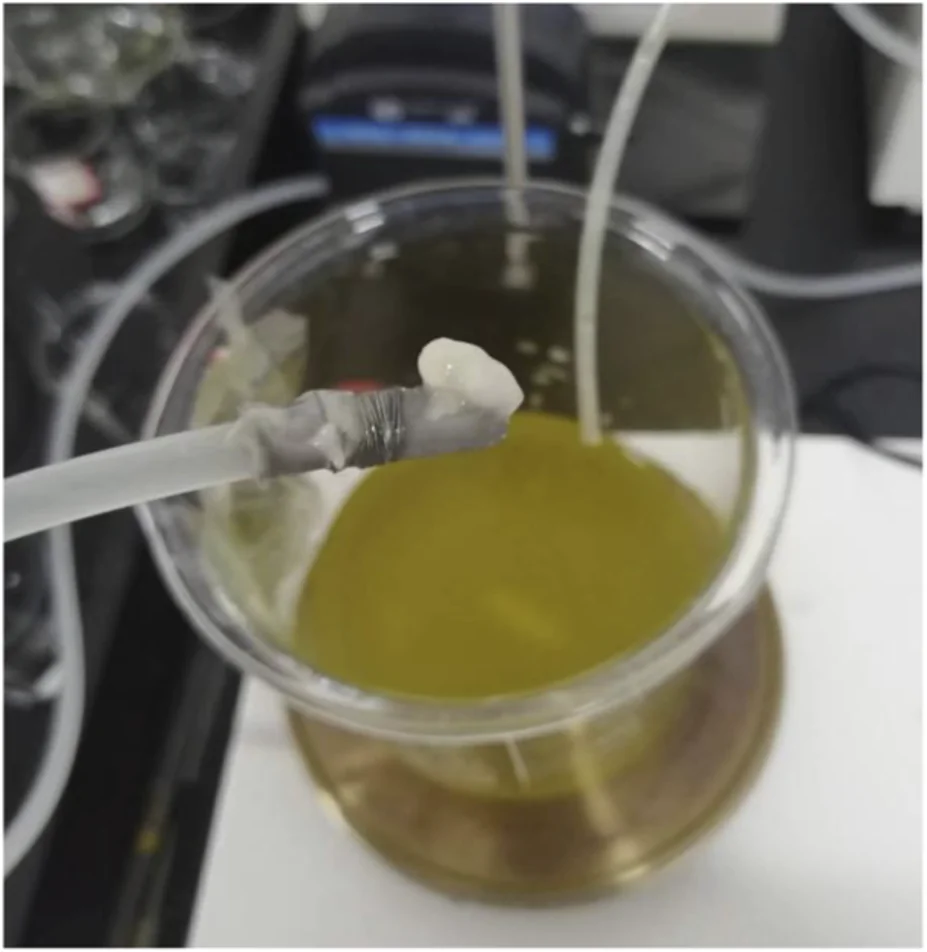
Fiber hanging phenomenon at the pipeline inlet.

Pulp fiber concentration exerts a remarkable influence on system stability. When the concentration exceeded 0.7%, untreated softwood, hardwood and hemp fibers failed to disperse uniformly in water, which made the detection unable to proceed normally. For softwood pulp, concentration above 0.4% would cause severe fiber hanging at the filter inlet after long-time operation (Figure 4). A large number of microbubbles were generated and flowed into the cuvette, enhancing light scattering and resulting in falsely high absorbance data.

Considering fiber types and morphological characteristics comprehensively, the pulp fiber concentration was finally set as 0.2% for routine tests to eliminate the adverse impact of fiber hanging. It should be noted that the concentration can be properly adjusted according to fiber categories and beating degrees when exploring the effects of pulp concentration or beating degree on dye adsorption.

#### 3.2.3 Selection and control of dye concentration

Dye concentration is a key factor affecting both adsorption behavior and spectral detection. When the concentration of brown dye was higher than 100 mg/L, the maximum absorption peak at 475 nm became flat by using a standard 10 mm optical path cuvette, which made effective measurement impossible. For high-concentration tests, cuvettes with shorter optical paths (1 mm or 0.5 mm) are required. Besides, different UV-Vis spectrometers have different designed maximum absorbance ranges, so the correlation between dye concentration and absorbance should be taken into account.

For high-concentration dye solutions, spectral red shift or blue shift can be observed, which is an important indicator of dye molecular aggregation. Dye molecules exist in monodisperse state at low concentrations. With the increase of concentration, the ionization degree decreases and molecular collision frequency rises. Dye molecules gradually aggregate into dimers, trimers and even larger clusters. Excessive aggregation will hinder the diffusion of dye molecules from fiber surface to interior, and further affect the surface color and color fastness of dyed fibers.

### 3.3 Calculation of the adsorption capacity of brown dye on pulp fibers

**FIGURE 5:**
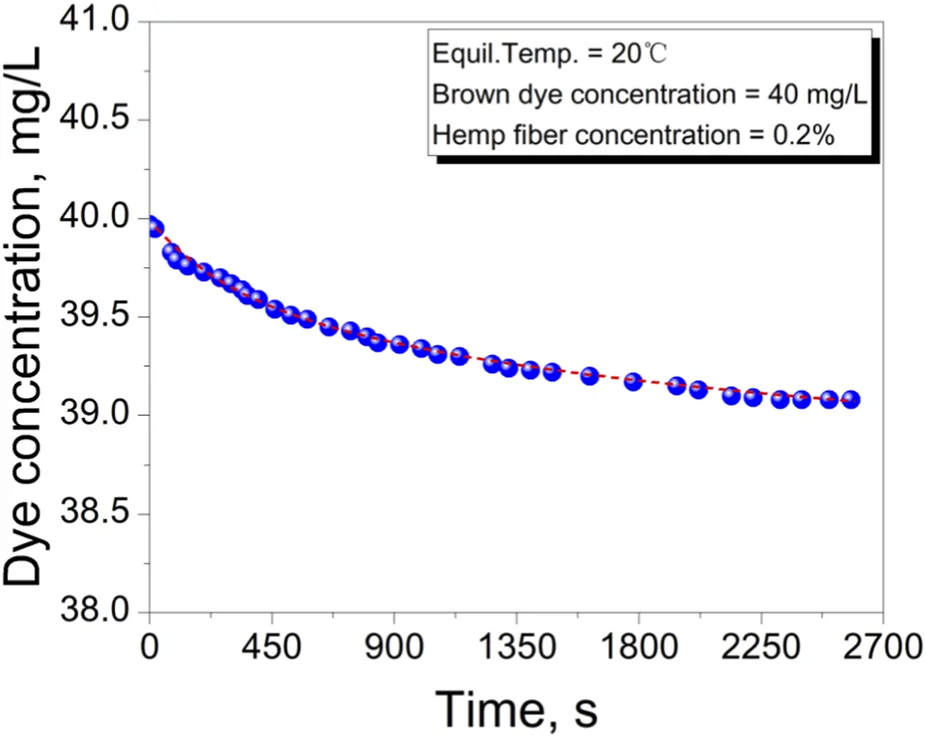
Relationship between absorbance value of brown dye solution and the adsorption time.

Figure 5 shows the relationship between absorbance at 475 nm and adsorption time, obtained by online kinetic monitoring after hemp pulp fibers (beating degree: 24.5 °SR) were added to the brown dye solution. As shown in the figure, before 2,500 s, the absorbance of the brown dye gradually decreased with increasing adsorption time. After 2,500 s, the absorbance remained essentially unchanged and reached a stable state.

The adsorption capacity of brown dye on pulp fibers was calculated from the change in dye concentration before and after the addition of pulp fibers to the brown dye solution. According to Equation 2, the mass of brown dye adsorbed per unit oven-dry mass of pulp fiber was obtained.

$$C_{s,t}=\frac{\left(C_{0}-C_{l,t}\right)\cdot V}{m}$$ (2)

where *C*_*s,t*_ is the adsorption capacity of brown dye at time t (mg/g); *C*_*0*_ and *Cl*_*,t*_ are the initial concentration and the concentration at time t of the brown dye solution, respectively (mg/L); *V* is the volume of the brown dye solution (L); and *m* is the oven-dry mass of pulp fibers in the beaker (g).

By combining Equations 1,2, the formula for calculating the adsorption capacity of brown dye can be obtained.

$$C_{s,t}=\frac{\left(A_{0}-A_{l,t}\right)\cdot V}{0.0029m}$$ (3)

Here, *A*_*0*_ is the initial absorbance of the dye solution, and *A*_*s,t*_ is the absorbance of the dye solution at adsorption time t. The adsorption rate of dye onto pulp fibers during the adsorption process can be calculated according to Equation 3.

**FIGURE 6:**
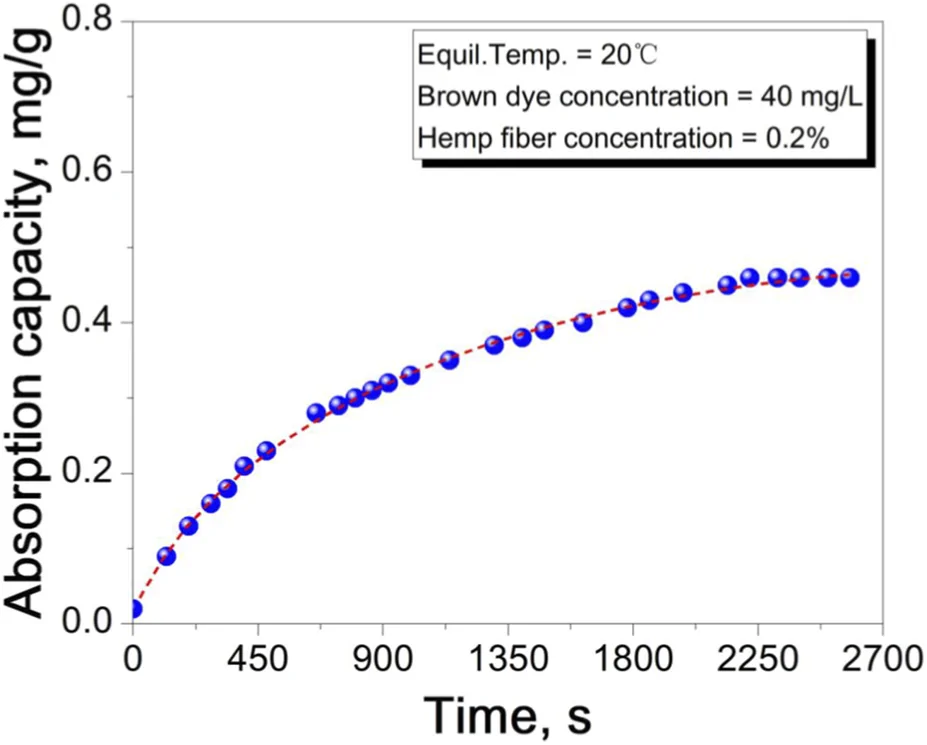
Relationship between adsorption capacity and adsorption time of hemp fiber for brown dye.

Accordingly, the change in absorbance can be determined using Equation 3. Figure 6 shows the variation in dye adsorption capacity of pulp fibers as a function of time.

### 3.4 Effect of the initial state of pulp fibers on the adsorption of brown dye

**FIGURE 7:**
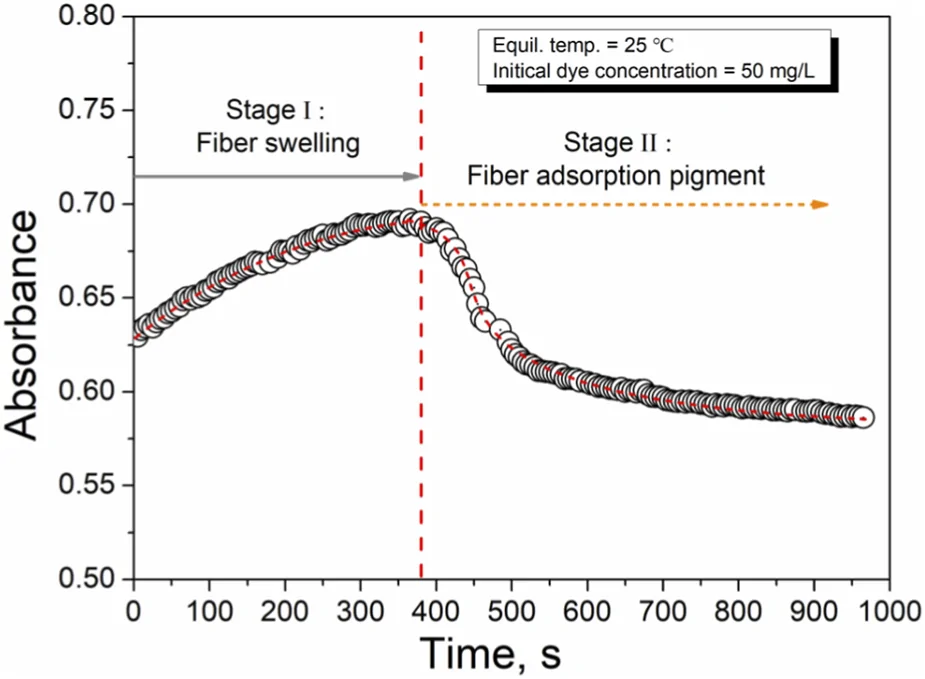
Adsorption process of brown dye by flake pulp fibers.

In the sheet-like hemp pulp fiber/brown dye aqueous system, the variation in solution absorbance at 475 nm with time after the addition of pulp fibers was monitored online using the kinetic monitoring platform, as shown in Figure 7. As can be seen, within 0–380 s, the absorbance of the brown dye increased slowly at first. This indicates that the total amount of solvent water in the solution decreased during this stage, resulting in an increase in brown dye concentration. Cellulose is a linear natural polymer composed of D-glucose units linked by 1,4-β-glycosidic bonds. Fiber bundles contain many functional groups, such as hydroxyl and carboxyl groups. The hydrogen-bonding network formed by these hydrophilic groups plays an important role in the water absorption and water retention properties of fibers (Jiang et al., 2022; Kuang et al., 2014; Wei, 2022). It can therefore be inferred that the adsorption process in the sheet-like hemp pulp fiber/brown dye aqueous system can be divided into two stages. The first stage is fiber water absorption and swelling, in which water uptake dominates, while dye adsorption is secondary. Under the driving forces of hydrogen bonding and related interactions, water cluster molecules preferentially adsorb at the solid fiber interface rather than dye molecules. This causes the fibers to soften. Before a dynamic adsorption equilibrium is reached between water molecules and adsorption sites, no or only a small number of dye macromolecules diffuse to the fiber surface and become adsorbed. The second stage is fiber dye adsorption, in which dye adsorption becomes dominant, while water absorption plays a secondary role. When the adsorption time exceeded 380 s, the absorbance of the brown dye began to decrease continuously, indicating that dye molecules started to adsorb onto the surface of the swollen fibers and gradually diffused into the fiber channels and interfiber pores. These results suggest that, in the aqueous system, water cluster molecules and dye macromolecules exhibit competitive adsorption on the solid fiber surface.

**FIGURE 8:**
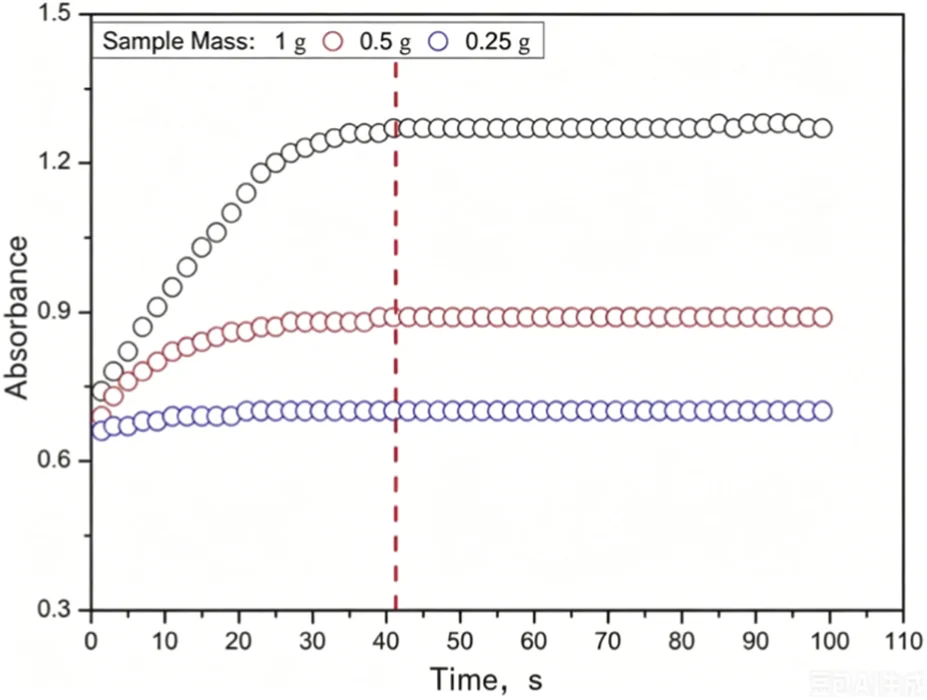
Desorption curve of dyed cigarette paper in aqueous solution.

To further elucidate the adsorption mechanism, we supplemented systematic solvent comparison experiments and desorption verification tests. Using the online UV-Vis monitoring platform established in this work, aqueous elution (desorption) assays were carried out on the as-prepared brown cigarette papers. The results demonstrated that the dye underwent nearly complete desorption within approximately 40 s (Figure 8), providing strong evidence that the interaction between the brown dye and pulp fibers is dominated by physical adsorption rather than chemical bonding.

**FIGURE 9:**
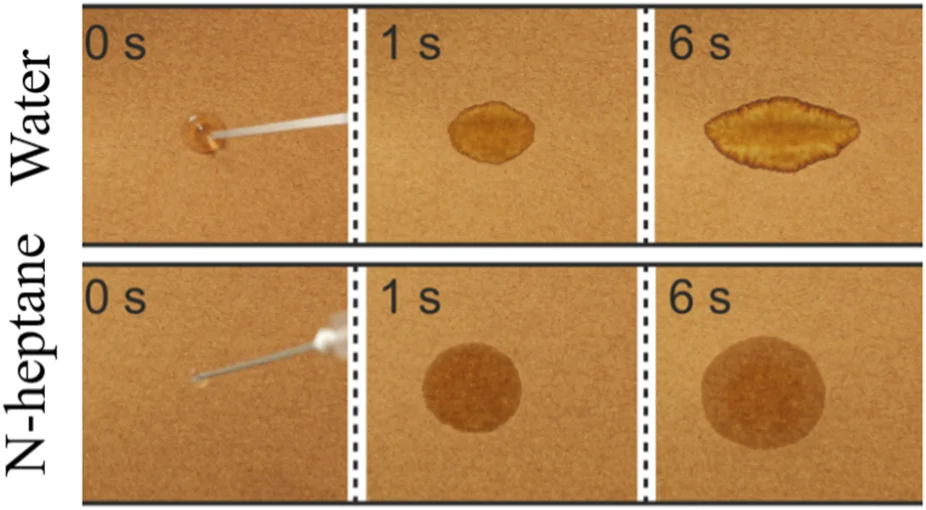
Morphological evolution of dyed brown paper surface after contact with water and n-heptane droplets.

Furthermore, comparative experiments using polar water and nonpolar n-heptane were designed to verify the competitive adsorption effect of water molecules as shown in Figure 9. When a 50 μL water droplet was applied to the dyed paper, water molecules disrupted the original inter-fiber hydrogen bonds and reconstructed a loose, interconnected pore structure. Water clusters interacted with the aggregated dye molecules, disassembling large dye aggregates into small molecules and triggering pronounced lateral dye diffusion along with the formation of color accumulation rings. In contrast, nonpolar n-heptane only penetrated into the pore interstices, without disrupting the hydrogen bond network or inducing dye diffusion. The distinct difference in behavior between water and n-heptane fully confirms that polar water molecules preferentially occupy the hydroxyl sites on the fiber surface and compete with dye molecules for adsorption positions. This competitive adsorption behavior accounts for the induction period and dynamic mass transfer characteristics observed during the adsorption process.

**FIGURE 10:**
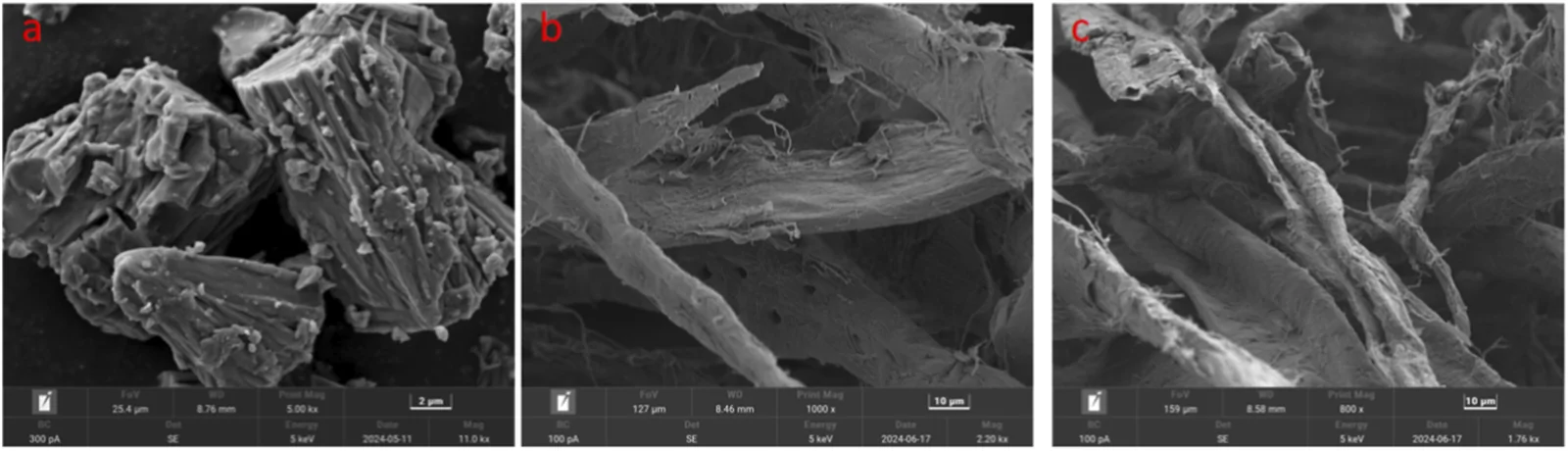
SEM images. **(a)** Pure dye; **(b)** Pristine pulp fibers; **(c)** Pulp fibers after dyeing.

The microstructures of the dye and the surface morphologies of pulp fibers before and after adsorption were characterized via scanning electron microscopy (Figure 10). No conspicuous deposition of dye particles was observed on the fiber surfaces at the microscale. This morphological observation is consistent with a physisorption mechanism at the molecular level, and aligns well with the conclusions drawn from our adsorption kinetic analysis and aqueous desorption tests.

### 3.5 Effect of dye concentration on dye adsorption by pulp fibers

**FIGURE 11:**
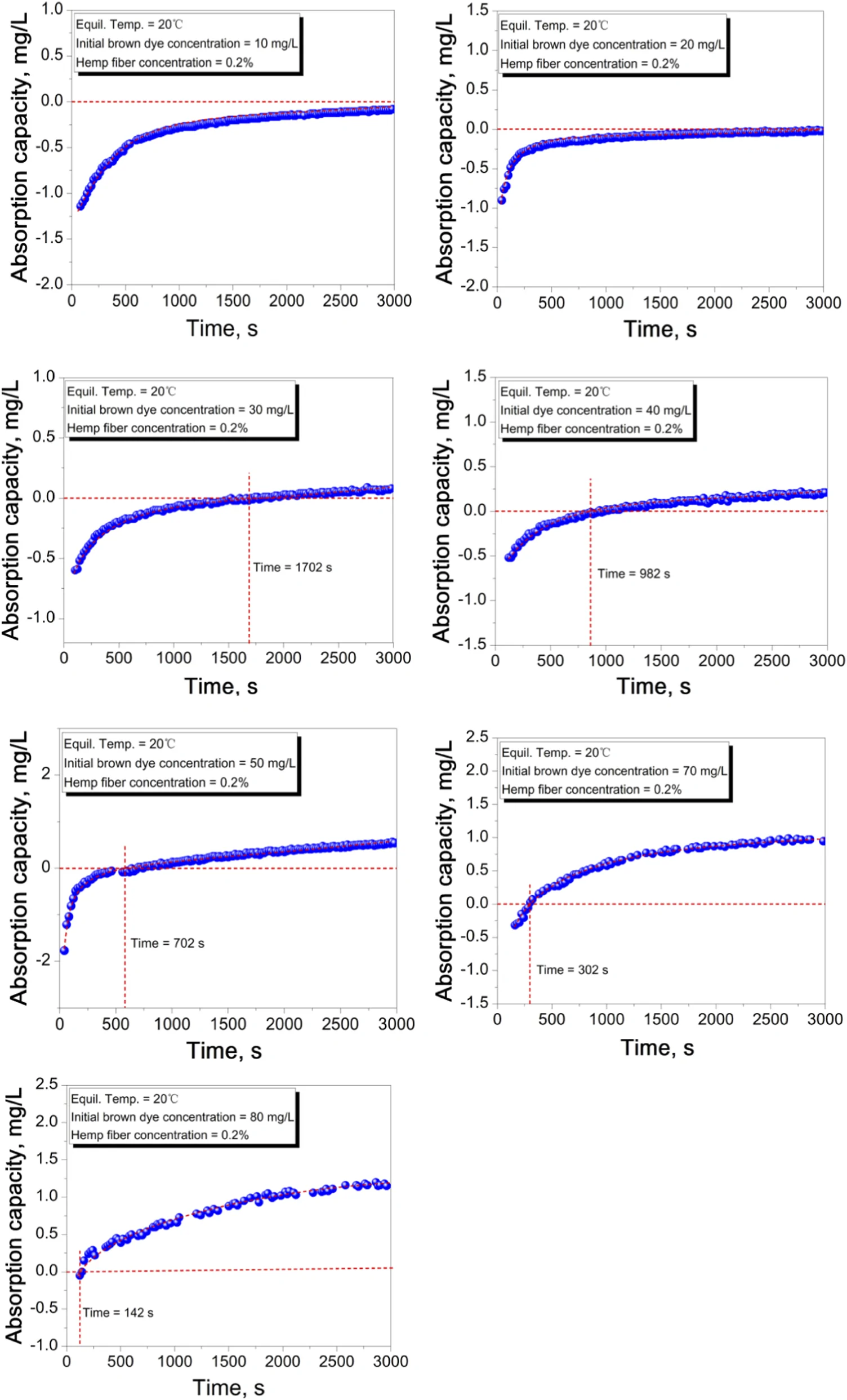
Variation of dye concentration in solution with adsorption time under different concentrations of brown dye.

Figure 11 shows the change in dye concentration with adsorption time in hemp pulp suspensions at different initial brown dye concentrations. As shown, dye concentration had a significant effect on the adsorption behavior of insufficiently swollen hemp pulp fibers. When the brown dye concentration was below 30 mg/L, the calculated dye concentration remained negative within 0–50 min of adsorption. This indicates that the dynamic dye concentration in the system was consistently higher than the initial dye concentration. In other words, when the dye concentration was below 30 mg/L, almost no dye adsorption occurred on the hemp pulp fibers. Once the dye concentration exceeded 30 mg/L, dye adsorption began to occur on the fiber surface. With increasing dye concentration, both the adsorption capacity and the adsorption rate increased.

**FIGURE 12:**
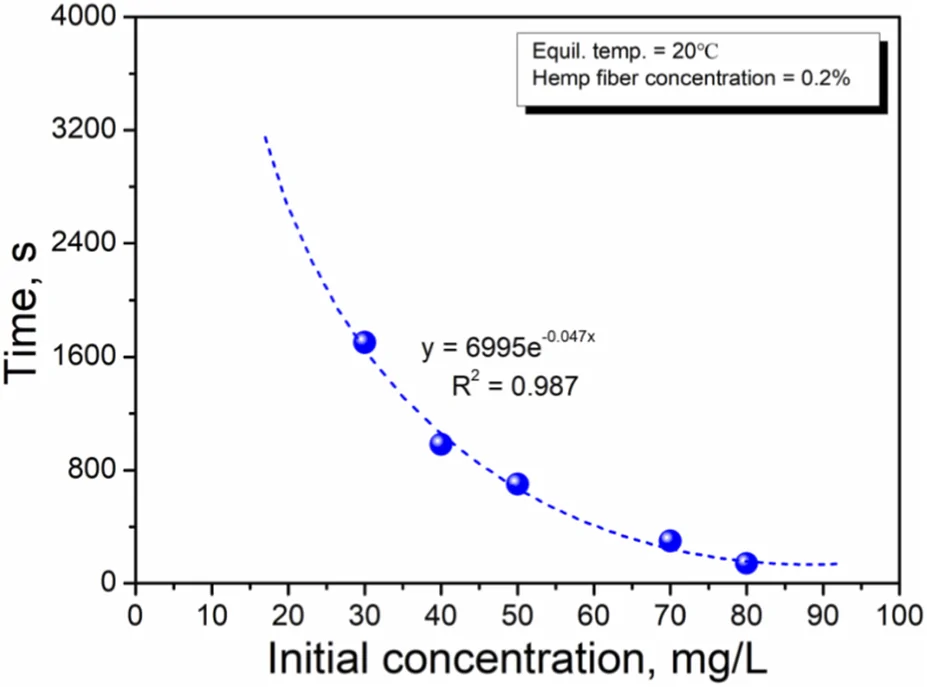
Actual required time for initial adsorption of pulp fibers at different initial dye concentrations.

The relationship between the initial dye concentration and the time required for pulp fibers to initiate dye adsorption is shown in Figure 12. As the dye concentration increased, the time required for the onset of dye adsorption decreased. A good mathematical relationship was observed between the initial concentration and the required time for the initiation of adsorption (y = 6,998*e^−0^·^047^ˣ, R^2^ = 0.987). This model indicates that, at lower dye concentrations, the initial coloration time increases sharply, which is unfavorable for pulp dyeing. In contrast, higher dye concentrations markedly reduce the initial coloration time.

**FIGURE 13:**
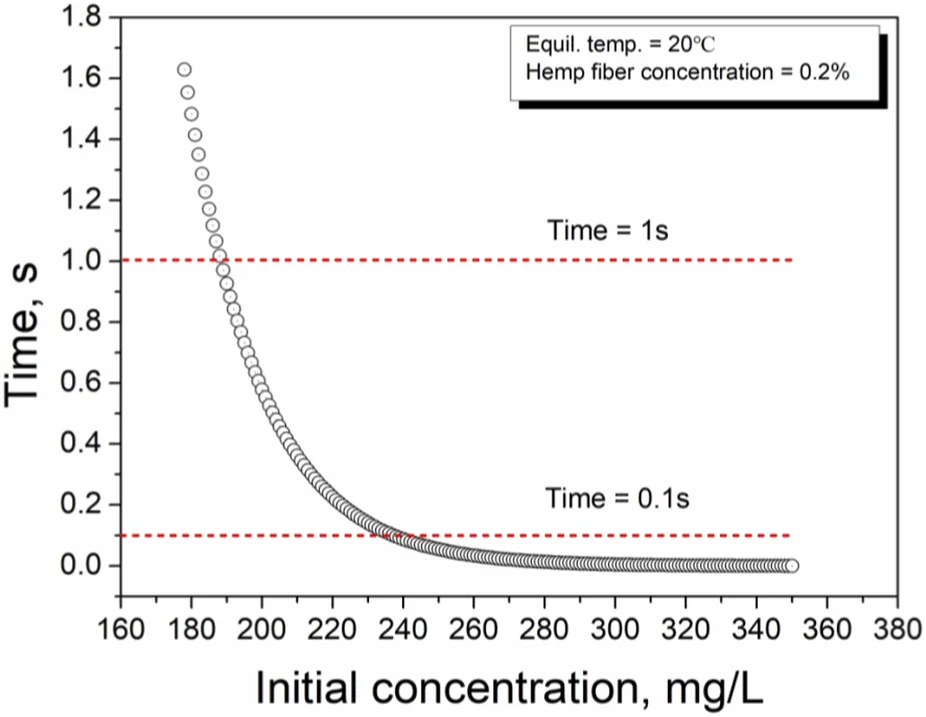
Theoretical required time for initial adsorption of pulp fibers at different initial dye concentrations.

As shown in Figure 13, based on the theoretical curve derived from this model, when the brown dye concentration exceeded 189 mg/L, the initial coloration time was shorter than 1 s. When the concentration exceeded 336 mg/L, the initial coloration time was shorter than 1 ms. In many modern colored cigarette paper workshops, online dyeing processes are used, where the coloration time is extremely short. Therefore, it is necessary to substantially increase the initial dye concentration in order to reduce the time required for pulp fibers to achieve initial adsorption.

**FIGURE 14:**
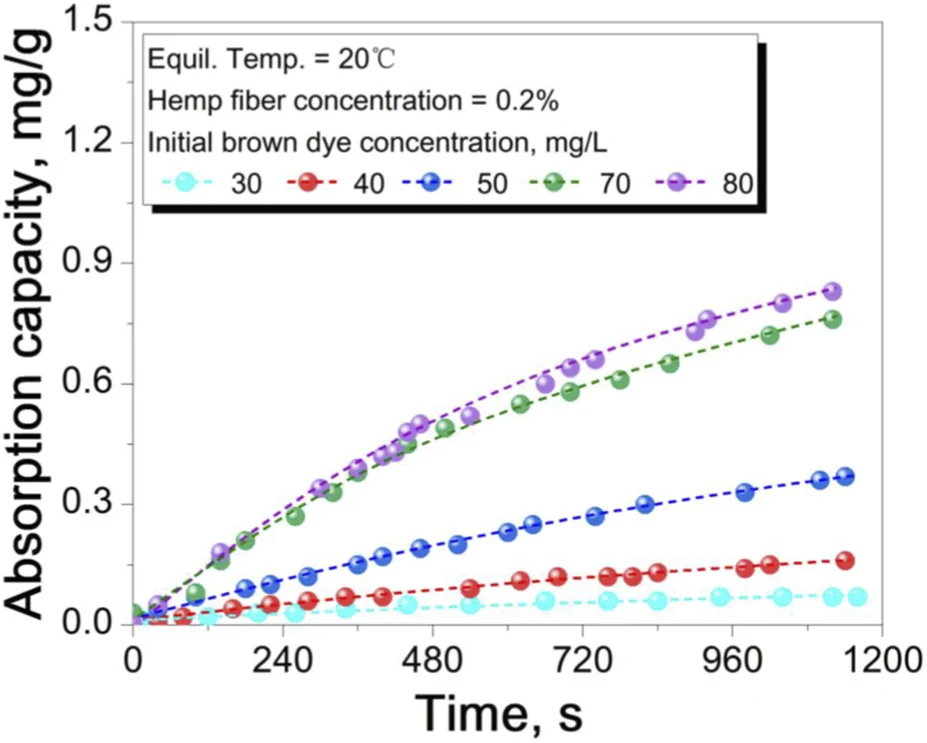
Variation in adsorption capacity of different concentrations of brown dye on hemp pulp fibers over time.

As discussed above, Stage I was dominated by fiber water absorption and swelling, whereas Stage II was dominated by dye adsorption by pulp fibers. To further clarify the adsorption behavior of brown dye on hemp pulp fibers during Stage II, this study examined the variation in dye adsorption capacity with time at different brown dye concentrations. The results are shown in Figure 14. At a given brown dye concentration, the adsorption capacity of the pulp fibers increased with adsorption time. At a fixed adsorption time, increasing the dye concentration effectively enhanced the adsorption capacity of the fibers.

**FIGURE 15:**
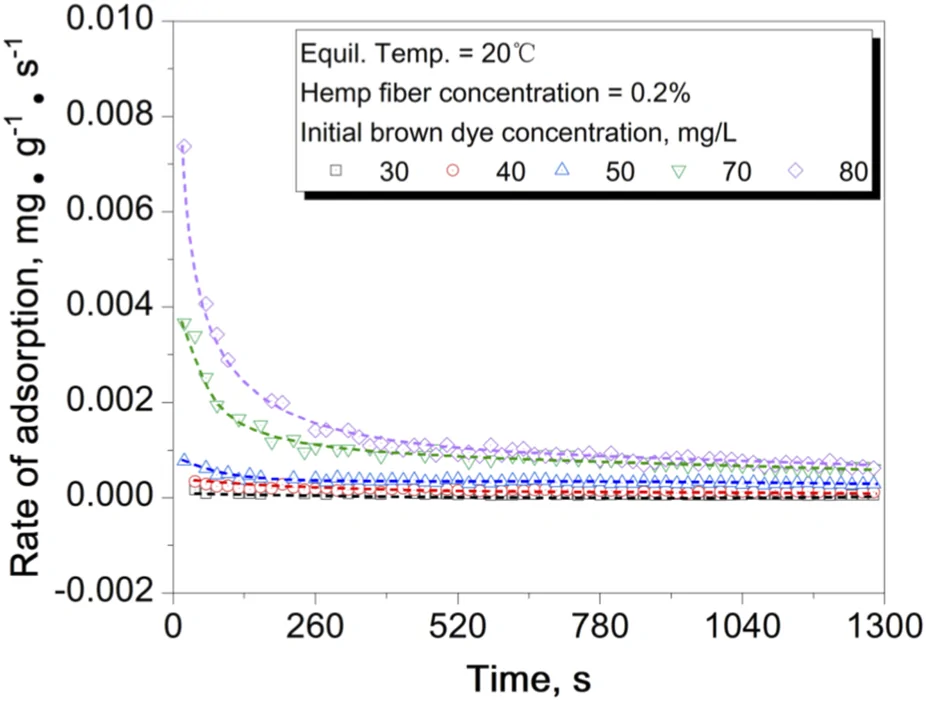
Variation in adsorption rate of different concentrations of brown dye on hemp pulp fibers over time.

Figure 15 shows the change in dye adsorption rate on fibers with time at different brown dye concentrations. As shown, the adsorption rate in the initial stage was markedly higher than that in the later stage. This is because, in the early stage, more adsorption sites were available on the fiber surface, allowing dye molecules to occupy these sites more readily. As adsorption proceeded, the dye gradually diffused into the interior of the fibers. Owing to steric hindrance, the adsorption rate gradually decreased until the available internal adsorption sites were occupied by dye molecules. A higher dye concentration produced a larger concentration gradient between the solution and the fiber surface, and this driving force significantly promoted dye diffusion toward the fibers.

These results indicate that, in the dyeing process, the effects of dye concentration and adsorption (residence) time on dye adsorption by pulp fibers should be fully considered. Appropriately increasing dye concentration and extending dyeing time can improve the adsorption performance of fibers toward the dye.

To obtain a more rigorous understanding of the variation in dye concentration on the pulp fiber surface under different dye concentrations, the pseudo-first-order was employed in this study to investigate the adsorption process of brown dye on hemp pulp fibers. The pseudo-first-order kinetic model assumes that the adsorption of dye onto fibers is a physical adsorption process, and that the adsorption behavior is mainly controlled by concentration diffusion (Brandani, 2021; Hong et al., 2021).

The pseudo-first-order kinetic model is expressed as follows:

$$q_{t}=q_{e}\left(1-e^{-k_{1}t}\right)$$ (4)

where *q*_*t*_ is the adsorption capacity at time t (mg/g), *q*_*e*_ is the equilibrium adsorption capacity (mg/g), *K*_*1*_ is the pseudo-first-order rate constant (s^−1^), and t is time (s). Rearrangement yields the first-order kinetic equation:

$$\ln\left(q_{e}-q_{t}\right)=\ln q_{e}-k_{1}t$$ (5)

The experimental data were fitted using the pseudo-first-order model, and the corresponding kinetic parameters for the adsorption of brown dye onto hemp pulp fibers are listed in Table 1. As shown in the table, the pseudo-first-order kinetic model exhibited good linearity (0.990 ≤ R^2^ ≤ 0.997). Therefore, the adsorption behavior of hemp pulp fibers toward brown dye can be described by the pseudo-first-order kinetic model, indicating that the adsorption process is predominantly physical adsorption.

**TABLE 1:** Fitting parameters of the adsorption kinetics model for brown dye on hemp pulp fibers.

Brown dye concentration (mg/L)	Pseudo-first-order kinetic model		
	q_e_ (mg/g)	K_1_ (s^-1^)	R^2^
30	0.0759	0.0022	0.9837
40	0.2223	0.0011	0.9927
50	0.6027	0.0008	0.9941
70	0.9738	0.0013	0.9944
80	1.1184	0.0012	0.9980

Based on the data for adsorption time and adsorption capacity of pulp fibers, the parameters in Equation 4 were fitted by the partial least squares method, and the optimal values of the parameters are listed in Table 1. By substituting the fitted optimal values in Table 1 into the pseudo-first-order kinetic equation, Equation 5, five fitted equations corresponding to five different brown dye concentrations were obtained, as shown in Table 2.

**TABLE 2:** Fitted equations for dye adsorption by pulp fibers at different brown dye concentrations.

Brown dye concentration (mg/L)	Fitting equation
30	$\ln\left(0.0759-q_{t}\right)=\ln q_{t}-0.0022t$
40	$\ln\left(0.2223-q_{t}\right)=\ln q_{t}-0.0011t$
50	$\ln\left(0.6027-q_{t}\right)=\ln q_{t}-0.0008t$
70	$\ln\left(0.9738-q_{t}\right)=\ln q_{t}-0.0013t$
80	$\ln\left(1.1184-q_{t}\right)=\ln q_{t}-0.0012t$

**FIGURE 16:**
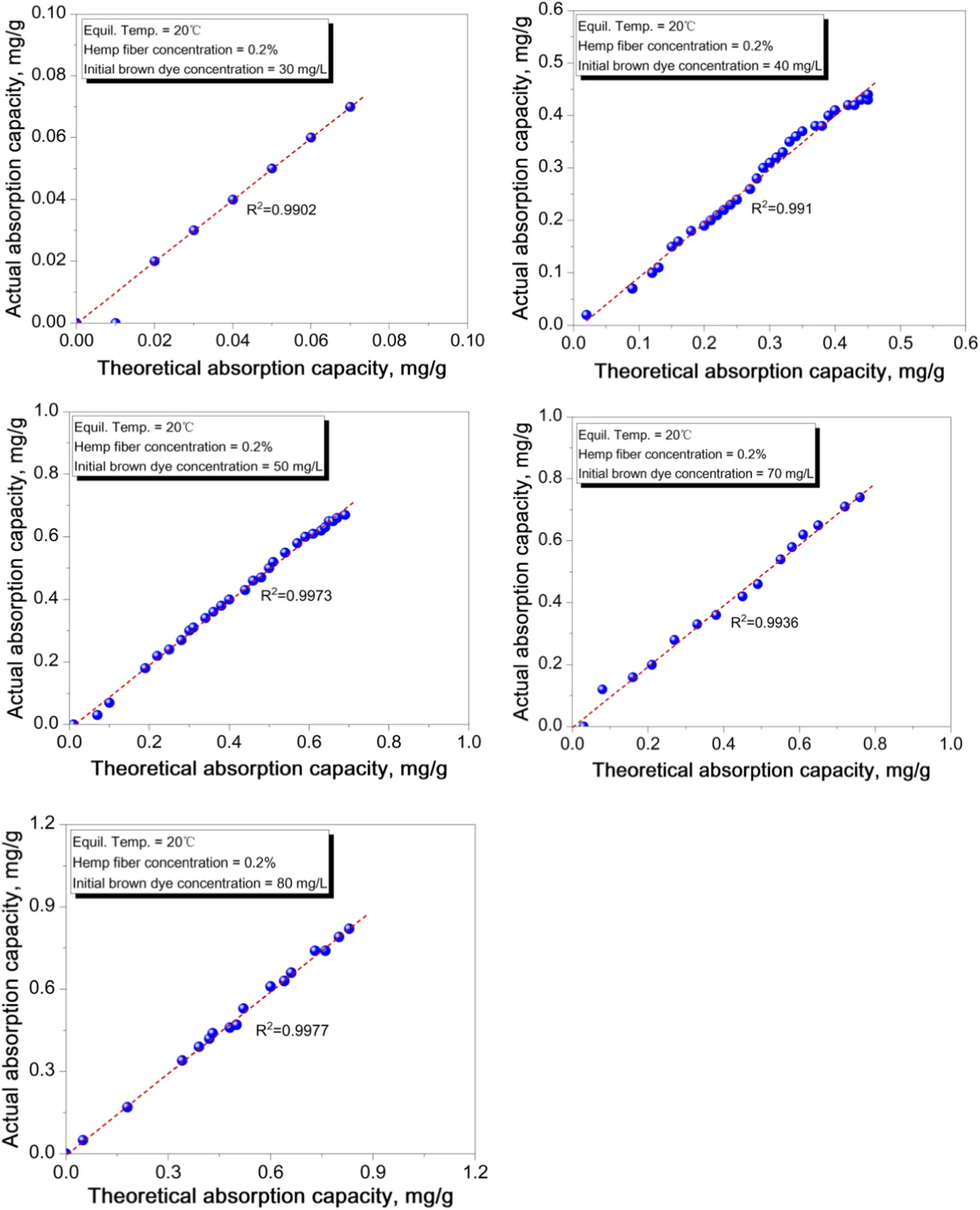
Relationship between the predicted adsorption values from the pseudo-first-order kinetic model and the experimentally measured adsorption values at different dye concentrations.

The theoretical equations listed in the table were used to calculate the theoretical adsorption capacities at brown dye concentrations of 30, 40, 50, 70, and 80 mg/L based on the pseudo-first-order kinetic model. These theoretical values were then fitted against the experimentally measured values, and the results are shown in Figure 16. As can be seen, good linear relationships were obtained for all fitted curves at different brown dye concentrations (0.9902 ≤ R^2^ ≤ 0.9971), indicating good agreement between the experimental and predicted values. Therefore, from the perspective of adsorption kinetics, the adsorption of brown dye onto hemp pulp fibers in brown dye solution is mainly governed by the concentration diffusion step. No electron sharing or electron transfer occurs between the dye molecules and the molecular chains of the pulp fibers. Hence, the adsorption process of brown dye on hemp pulp fibers can be well described by the pseudo-first-order kinetic model.

**FIGURE 17:**
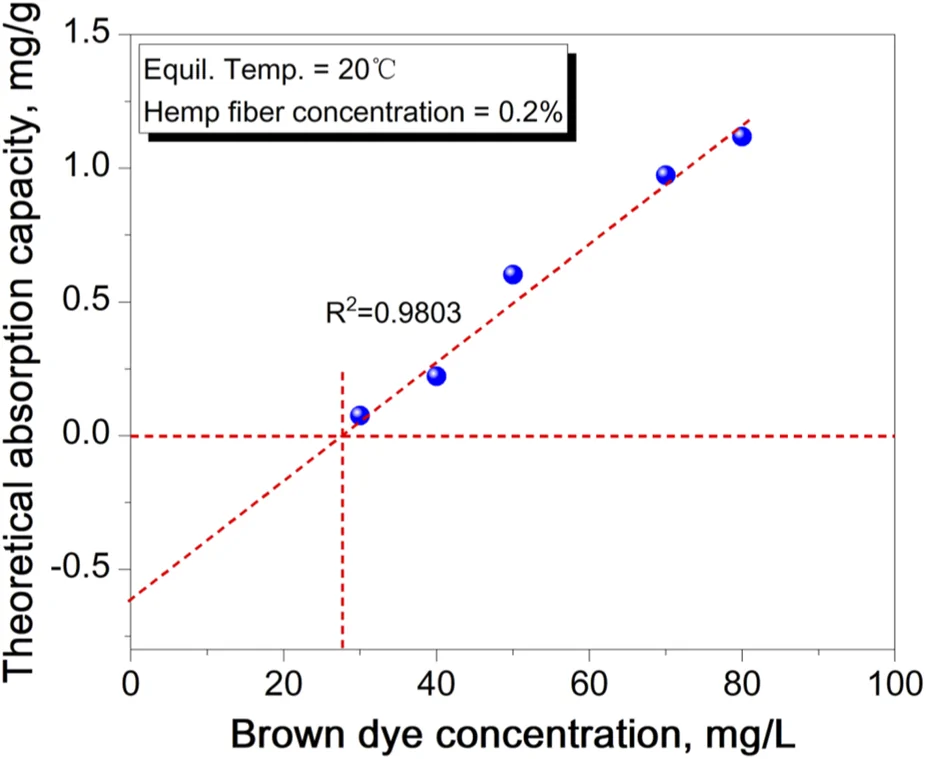
Relationship between equilibrium adsorption capacity and brown dye concentration.

Based on the data for brown dye concentration and equilibrium adsorption capacity (q_e_) in Table 1, the relationship between q_e_ and brown dye concentration was plotted, as shown in Figure 17. When the brown dye concentration was lower than 30 mg/L, the equilibrium adsorption capacity of dye on hemp pulp fibers was nearly zero. As discussed above, because competitive adsorption exists between dye molecules and water clusters on the fiber surface, the interaction between brown dye molecules and fibers is dominated by weak physical adsorption rather than chemical bonding. The adsorption of brown dye on pulp fiber surfaces follows the pseudo-first-order kinetic equation, indicating that the migration of brown dye molecules to the fiber surface is mainly controlled by the dye concentration gradient (Perdoch et al., 2022). When the brown dye concentration is too low, the concentration difference between the bulk solution and the fiber surface is too small to drive mass transfer of brown dye molecules to the fiber surface. It can also be seen from the figure that, with increasing brown dye concentration, the equilibrium adsorption capacity of dye on pulp fibers increased linearly. A good linear relationship was observed between dye concentration and q_e_ (R^2^ = 0.98). This provides a rational basis for regulating dye concentration and the equilibrium adsorption capacity on pulp fibers, and offers a practical approach for further enhancing paper coloration.

### 3.6 Adsorption behavior of dye on different types of pulp fibers

**FIGURE 18:**
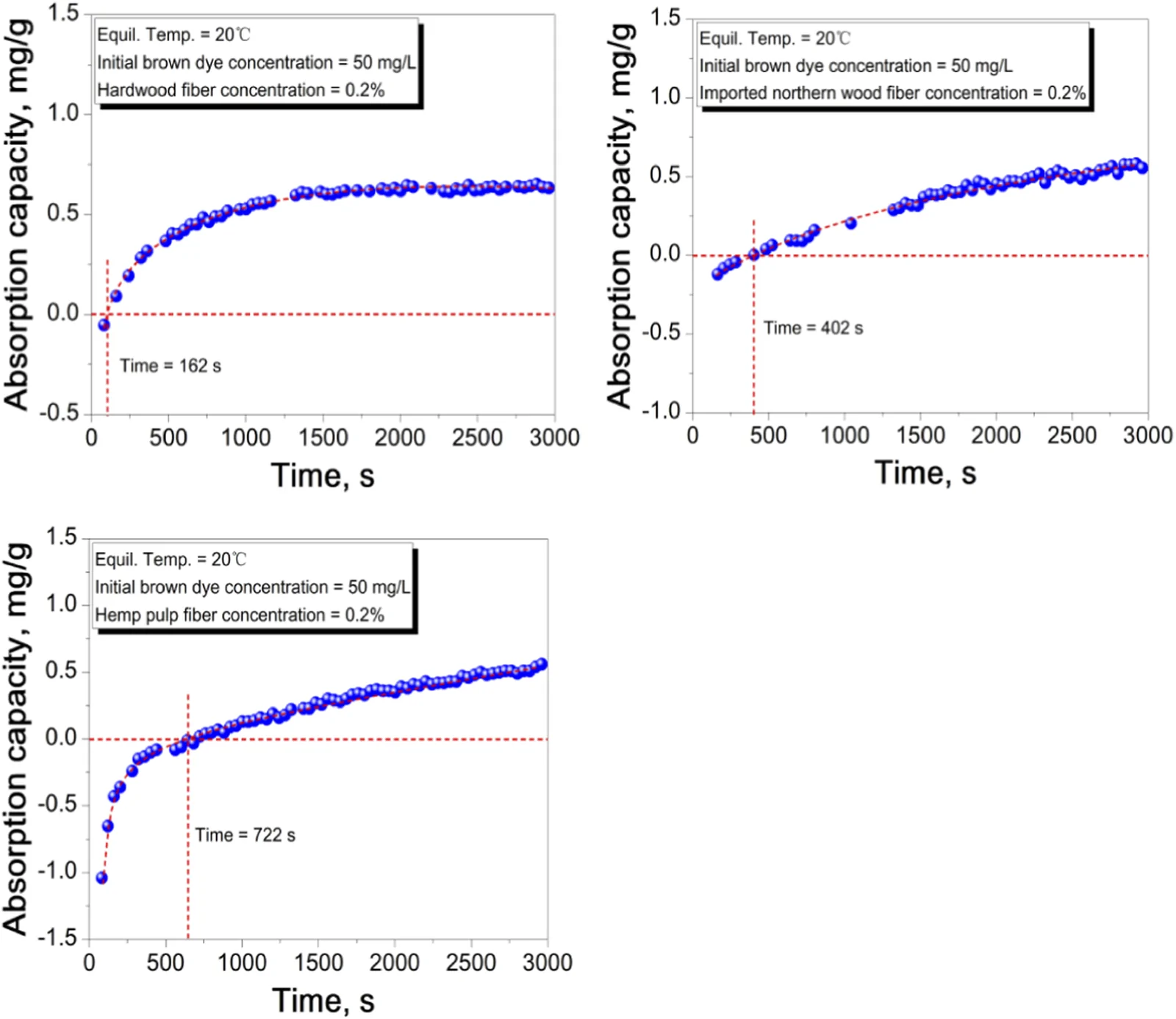
Adsorption of brown dye on different pulp fibers.

Cigarette paper is a soft and porous material with good air permeability. Its fibrous raw materials mainly include long-fiber pulp, short-fiber pulp, and hemp pulp (Huang, 2005; Ma and Cao, 2015). The fibrous raw materials used in cigarette papers of different grades and specifications vary in terms of pulp origin (imported or domestic) and fiber blending ratio. In the fiber composition of white cigarette base paper, long-fiber pulp mainly provides appropriate physical strength (Wei, 2022), short-fiber pulp contributes suitable bulk and air permeability (Zhang et al., 2021), and hemp pulp helps reduce woody odor during cigarette combustion and improve cigarette aroma (Ma and Cao, 2015). During the online dyeing of white base paper, fiber type affects the color difference and uniformity of colored cigarette paper. Figure 18 shows the adsorption behavior of brown dye on different pulp fibers. As can be seen, in terms of the initial adsorption time, short-fiber pulp exhibited the shortest initial adsorption time (T_o_), followed by long-fiber pulp (imported softwood) and hemp pulp. In terms of adsorption rate, short-fiber pulp showed a relatively high adsorption rate in the initial stage, which gradually decreased in the later stage. By contrast, the adsorption processes of long-fiber pulp and hemp pulp were relatively similar, and their adsorption rates remained nearly constant before 3,000 s. This can be attributed to the finer fiber size and larger specific surface area of short-fiber pulp, which facilitate faster dye adsorption. In terms of the time required to reach equilibrium, short-fiber pulp reached adsorption equilibrium within only 1,500 s, whereas long-fiber pulp and hemp pulp required more than 10,000 s.

Based on the data from the dye adsorption stage of pulp fibers (Stage II), the adsorption behavior of brown dye on different types of pulp fibers was analyzed using the pseudo-first-order model. The fitted parameters of the adsorption kinetic models are listed in Table 3. As shown in the table, the pseudo-first-order kinetic equation is applicable for describing the adsorption process of brown dye on different pulp fibers. The equilibrium adsorption capacities of softwood, hardwood pulp, and hemp pulp were 0.7312, 0.6327, and 0.7531 mg/g, respectively. These results indicate that different types of pulp fibers exhibit certain differences in their adsorption capacity toward brown dye, which in turn may affect the uniformity of paper coloration.

**TABLE 3:** Fitted parameters of the adsorption kinetic models for brown dye on different pulp fibers.

Types of pulp fibers	Pseudo-first-order kinetic models		
	q_e_ (mg/g)	K_1_ (s^-1^)	R^2^
Hardwood	0.6327	0.0024	0.9823
Softwood	0.7312	0.0006	0.9842
Hemp pulp	0.7531	0.0005	0.9906

### 3.7 Equilibrium adsorption capacity of pulp fibers with different types, beating degrees and blending ratios

This section preliminarily investigates the effects of fiber type, beating degree and fiber blending ratio on the equilibrium adsorption capacity of the dye. The brown dye used in this work is a soluble acid dye. Compared with basic dyes and reactive dyes, it exhibits lower affinity and poorer direct dyeing performance toward cellulosic fibers. A total of 15 pulp samples with combined gradient variables were tested, and the corresponding results are listed in Table 4.

**TABLE 4:** Equilibrium adsorption capacity of dye on pulp fiber samples with different fiber types, beating degrees and blending ratios.

Sample no.	Dye concentration (mg/L)	Beating degree (°SR)	Fiber type or blending ratio	Equilibrium adsorption capacity, mg/g (n = 3)*
1	50	19.8	Softwood pulp	0.133 ± 0.02
2		24.2		0.163 ± 0.02
3		31.6		0.175 ± 0.019
4		34.4		0.168 ± 0.021
5		37.7		0.177 ± 0.018
6	50	24.5	Hemp pulp	0.753 ± 0.022
7		31.6		0.712 ± 0.020
8		44		0.740 ± 0.021
9		61.7		0.834 ± 0.017
10		74.2		0.801 ± 0.021
11	50	-	Softwood: Hemp = 1:5	0.728 ± 0.018
12			Softwood: Hemp = 1:4	0.692 ± 0.022
13			Softwood: Hemp = 1:3	0.673 ± 0.018
14			Softwood: Hemp = 1:2	0.652 ± 0.023
15			Softwood: Hemp = 1:1	0.620 ± 0.017

* Data are presented as mean ± standard deviation (n = 3).

The influences of fiber type, beating degree and blending ratio on equilibrium dye adsorption capacity were preliminarily investigated. Fiber type dominates the adsorption performance, and hemp pulp shows superior dye adsorption behavior compared with softwood pulp. Variations in beating degree alter fiber morphology and pore structure, thus affecting adsorption efficiency, and excessive beating impairs the adsorption capacity of hemp pulp. For blended softwood and hemp pulps, the equilibrium adsorption capacity decreases gradually as the proportion of softwood pulp increases. These results offer theoretical guidance for the optimization of fiber blending and beating technology for colored cigarette paper.

## 4 Conclusion

This study systematically investigated the adsorption behavior of brown natural dye onto three typical pulp fibers (hardwood, softwood, and hemp pulp) using a self-built online UV-Vis spectral monitoring system, filling the gap of real-time kinetic data for the online dyeing process of colored cigarette paper. The adsorption process presents distinct two-stage characteristics, consisting of a water absorption-swelling induction period and a subsequent dye adsorption stage. A competitive adsorption mechanism between water molecules and dye macromolecules is proposed to account for the induction period, and the induction time shows an exponential negative correlation with initial dye concentration (R^2^ = 0.987). Within the concentration range of 30–80 mg/L, the equilibrium adsorption capacity increases linearly with initial dye concentration. The pseudo-first-order kinetic model delivers the best fitting performance for all experimental systems, indicating that the adsorption is dominated by physisorption and controlled by mass transfer diffusion. Fiber type exerts a significant influence on adsorption performance: hardwood pulp have the fastest initial adsorption rate and reach equilibrium at approximately 1,500 s, while softwood and hemp pulp require more than 10,000 s to achieve equilibrium. At 50 mg/L dye concentration, the qe values of hardwood, imported softwood and hemp pulp are 0.6327, 0.7312 and 0.7531 mg/g, respectively. These findings can provide theoretical guidance and data support for fiber formula optimization and online dyeing process regulation of colored cigarette paper.

## Data Availability

The original contributions presented in the study are included in the article/supplementary material, further inquiries can be directed to the corresponding authors.
